# Health outcomes after national acute sleep deprivation events among the American public

**DOI:** 10.1172/jci.insight.195344

**Published:** 2025-12-23

**Authors:** Neil J. Kelly, Rahul Chaudhary, Wadih El Khoury, Nishita Kalepalli, Jesse Wang, Priya Patel, Irene N. Chan, Haris Rahman, Aisha Saiyed, Anisha N. Shah, Colleen A. McClung, Satoshi Okawa, Seyed Mehdi Nouraie, Stephen Y. Chan

**Affiliations:** 1 Center for Pulmonary Vascular Biology and Medicine, Pittsburgh Heart, Lung, and Blood Vascular Medicine Institute, and; 2 Heart and Vascular Institute, University of Pittsburgh School of Medicine and University of Pittsburgh Medical Center, Pittsburgh, Pennsylvania, USA.; 3 Pittsburgh VA Medical Center, Pittsburgh, Pennsylvania, USA.; 4 Wellesley College, Wellesley, Massachusetts, USA.; 5 University of California, Davis, Davis, California, USA.; 6 Translational Neuroscience, Department of Psychiatry,; 7 Department of Computational and Systems Biology,; 8 McGowan Institute for Regenerative Medicine, and; 9 Division of Pulmonary, Allergy, and Critical Care Medicine, Department of Medicine, University of Pittsburgh School of Medicine and University of Pittsburgh Medical Center, Pittsburgh, Pennsylvania, USA.

**Keywords:** Genetics, Public Health, Epidemiology, Genetic risk factors, Population health research

## Abstract

**BACKGROUND:**

Sleep is increasingly recognized as essential to human health, yet the adverse health consequences of acute sleep deprivation are unknown. We hypothesized that acute sleep deprivation is associated with health outcomes and modulated by sleep-associated genotypes.

**METHODS:**

Locally estimated scatterplot smoothing (LOESS) was performed on sleep estimates from Fitbit users (*n* = 14,681) between June 1, 2016, and July 1, 2022. Dates when population minutes slept were less than the 90% confidence interval of the LOESS regression were named acute sleep deprivation events (ASDEs). Phenome-wide disease incidence among the All of Us Research Program population (*n* = 287,012) in the 10 days after ASDE was compared with a preceding reference period by McNemar’s test. Circadian rhythm–associated and sleep duration–associated SNPs were screened to identify genotypes associated with shorter ASDE sleep duration. Influences of sleep and circadian genotype on post-ASDE influenza risk were modeled using binomial family generalized estimating equations.

**RESULTS:**

We identified 32 ASDEs spanning major national events. A phenome-wide screen found increased risk of influenza (odds ratio = 1.54 [1.40, 1.70], *P* = 1.00 × 10^–18^) following ASDEs. Fifty-six SNPs were associated with decreased sleep duration on ASDEs. Higher quantiles of ASDE-related SNP genotype burden were associated with less ASDE sleep duration and a greater risk of influenza-associated health care visits.

**CONCLUSION:**

Major national events are associated with acute sleep deprivation and greater influenza risk, which is amplified by sleep genotypes. These findings should inform public health vigilance surrounding major national events.

**FUNDING:**

WoodNext Foundation; NIH grants T32HL129964, K08ES037420, R01HL124021, R01HL122596, and R01HL151228; American Heart Association grants 24SFRNCCN1276089 and 24SFRNPCN1280228; and the United Therapeutics Jenesis Innovative Research Awards, the Pulmonary Hypertension Association, the McKamish Family Foundation, the Hemophilia Center of Western Pennsylvania, and the Institute for Transfusion Medicine.

## Abstract 2

Influenza risk is increased in accordance with sleep genotypes after national acute sleep deprivation events

## Graphical abstract

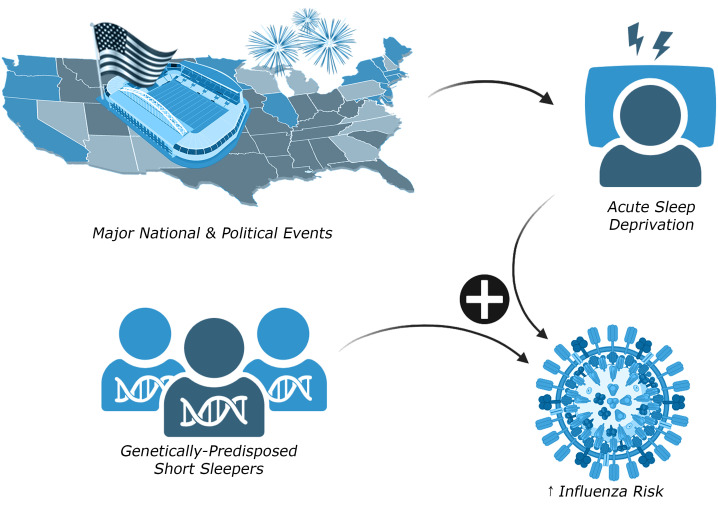

## Introduction

Sleep is essential and foundational for human health (1). Yet insufficient sleep duration is a common problem affecting nearly one-third of all US adults, which some studies suggest is becoming more pronounced over time (2, 3). The driving causes behind sleep deprivation are numerous, spanning individual, career, social, and cultural factors (1, 4).

Chronic sleep deprivation has been linked to myriad negative health consequences, including neurocognitive impairment (5), mental health and behavioral disorders (6), altered metabolism (7), immune dysregulation (8, 9), cardiovascular disease (10, 11), and all-cause mortality (12). Contrasted with chronic sleep deprivation, our understanding of the health impacts of acute sleep deprivation is limited. Based on small studies in laboratory settings, acute sleep deprivation is thought to have adverse effects on neurobehavioral, endocrine, and immune physiology (13). Recently, a small study detected substantial changes in the serum proteome after just a single night of sleep deprivation (14). However, the impacts of these transient changes on human disease are largely unknown. In part, this knowledge deficit may be explained by the difficulty of acute sleep deprivation studies in humans, which traditionally involve laboratory-based studies of healthy volunteers in controlled conditions. In other cases, studies are limited to small populations where sleep is monitored through diaries or wearable biosensors (15).

The All of Us (AoU) Research Program is a precision medicine initiative containing longitudinal health, genomic, and lifestyle data on a diverse cross section of more than 413,000 American participants (16). AoU also contains Fitbit-based daily sleep estimates for a subset of participants; these estimates track closely with previously reported estimates of sleep duration (17) and have revealed phenomic associations between chronic sleep patterns and disease (18). Here, we sought to leverage AoU to identify health outcomes associated with acute sleep deprivation. First, by Fitbit-based sleep monitoring, we identified dates, which coincided with major national events, that displayed acute sleep deprivation largely shared across the AoU subpopulations, which we describe as acute sleep deprivation events (ASDEs). Such a strategy bypassed any study confounders related to the heterogeneity of sleep deprivation patterns observed at the individual level across the calendar year. Second, in an unbiased phenome-wide analysis, we identified an increase in influenza incidence following population-level acute sleep deprivation. Finally, we incorporated a priori knowledge of sleep genomics to decouple the effects of sleep and circadian disturbances from other related stressors. By this approach, we found that acute sleep deprivation increases the risk of influenza, and that this risk is amplified by genotypes associated with shorter sleep duration.

## Results

### Background characteristics of the AoU populations.

**Figure 1:**
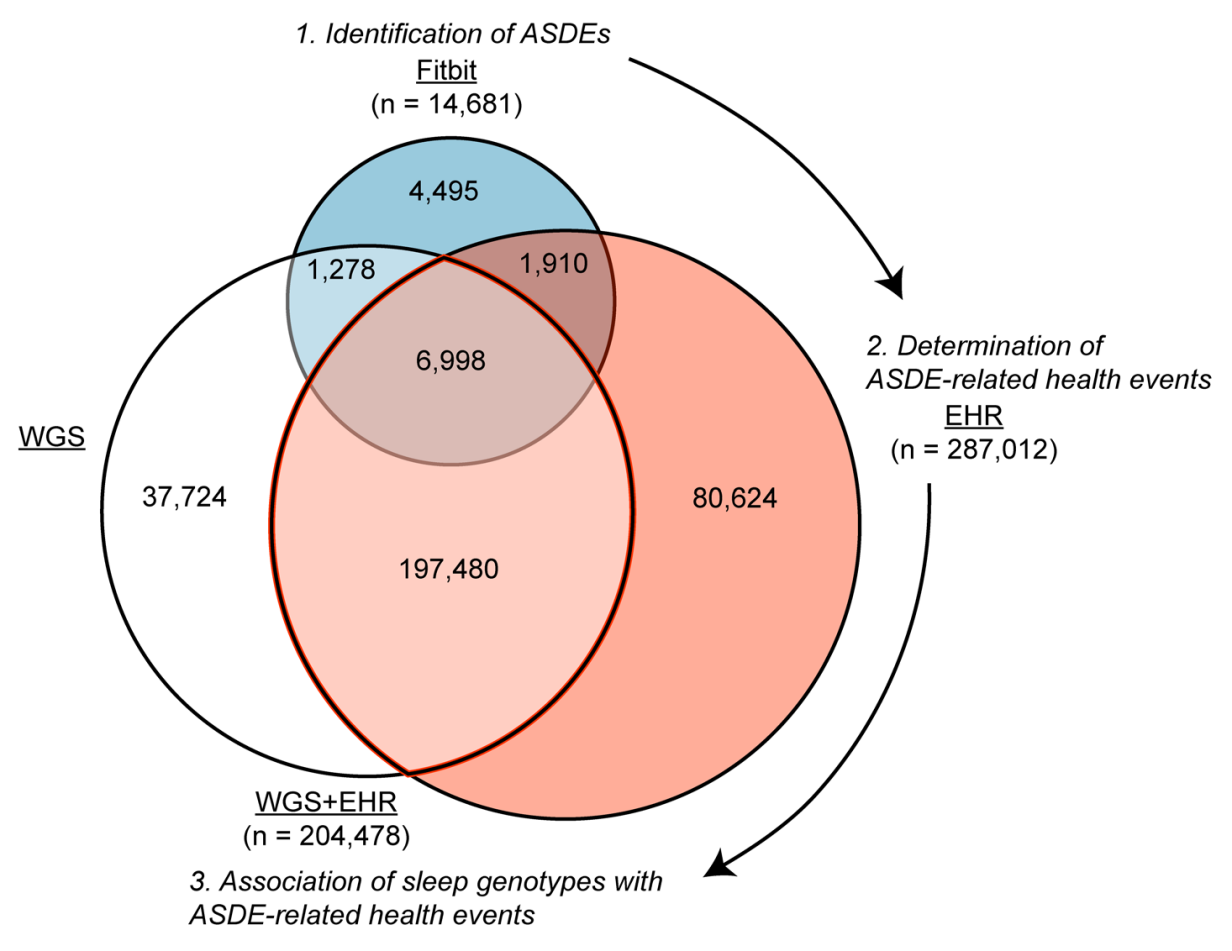
Subpopulations in the AoU populations and their utilizations. Venn diagram shows the partial overlaps of AoU populations with Fitbit data, EHR-linked data, and whole-genome sequencing (WGS). The Fitbit population was used to identify population-level acute sleep deprivation events (ASDEs), the EHR population was used to determine ASDE-related health events, and the overlapping WGS+EHR population was used to determine the association between sleep genotypes and ASDE-related health events.

The AoU population consisted of 413,457 participants. Subsets of the AoU population had linked electronic health record (EHR) data (*n* = 287,012 participants), whole-genome sequencing (*n* = 243,480 participants), and Fitbit data (*n* = 14,681 participants). Background characteristics of the AoU cohort and subgroups are provided in Table 1. These partially overlapping subgroups and their utilizations within the study framework are shown in Figure 1.

### Identification of ASDEs in the AoU population.

**Figure 2:**
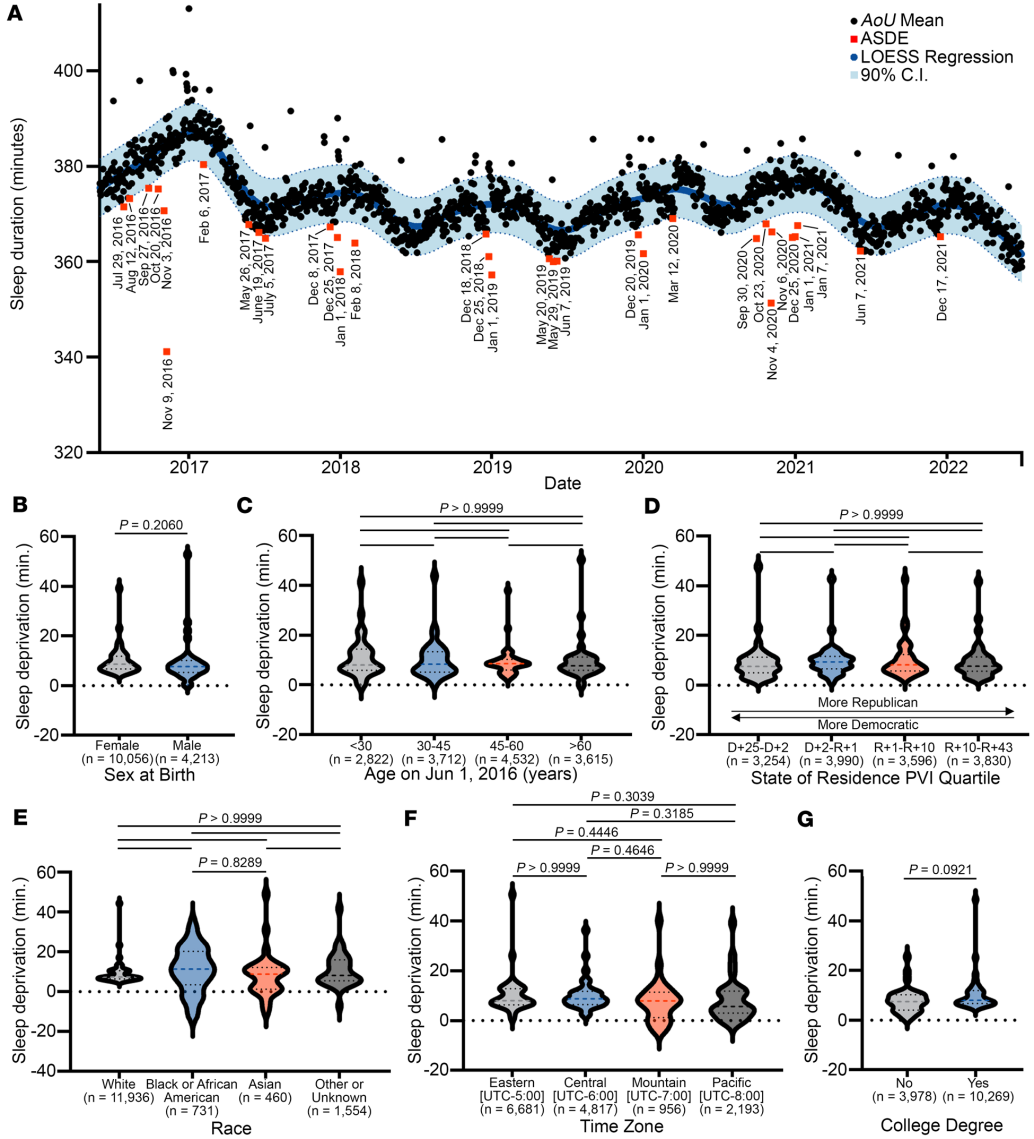
ASDEs across the AoU population, June 1, 2016, to July 1, 2022. (**A**) Daily average minutes slept were fit to a LOESS regression. Dates below the 90% confidence interval (C.I.) were named ASDEs (*n* = 14,681). (**B**–**G**) For each category of sex at birth (**B**), age range (**C**), state political lean (**D**), race (**E**), time zone (**F**), and education (**G**), LOESS smoothing was performed, and average minutes slept on ASDEs were subtracted from the LOESS fit to determine minutes of sleep deprivation. Dashed line on violin plots is median; dotted lines are interquartile range. Statistical comparisons were made by Mann-Whitney test (2 groups) or Kruskal-Wallis test with Dunn’s multiple-comparison test (3+ groups). D, Democratic; R, Republican; UTC, Coordinated Universal Time. *P* < 0.05 was considered statistically significant.

Using Fitbit-based estimates of average daily weekday minutes slept in the Fitbit population (*n* = 14,681 Fitbit users), we modeled expected daily minutes slept based on locally estimated scatterplot smoothing (LOESS) regression (Figure 2A). Next, to identify dates when average minutes slept were significantly less than expected, we identified 32 dates when the average minutes slept was less than the lower bound of the 90% confidence interval of the LOESS regression. We defined these dates as acute sleep deprivation events (ASDEs) and annotated each date based on temporal data from Google Trends (19), television viewership (20), and curated current events (21) (Table 2 and Supplemental Table 1; supplemental material available online with this article; https://doi.org/10.1172/jci.insight.195344DS1). The ASDEs corresponded to major national political occasions, holidays, media events, and athletic competitions. On these dates, the mean population sleep duration was 6 to 44 minutes lower than the LOESS regression (Table 2). We found no significant differences in sleep deprivation based on sex, age, state of residence, political leaning, race, time zone, and education level, suggesting that these ASDEs affect multiple segments of the population (Figure 2, B–G).

### ASDEs are associated with increased influenza health care visits.

In order to identify health outcomes associated with ASDEs, we conducted an unbiased phenome-wide analysis of the entire AoU population with linked EHR data (*n* = 287,012). To study the effect of ASDEs on health events, we first defined 10-day post-ASDE periods and preceding 10-day reference periods during which incident diagnoses were evaluated. Overlapping post-ASDE periods were pooled (to *N* = 23 ASDEs) such that the period concluded 10 days after the latest ASDE (Supplemental Figure 1). Reference periods (Supplemental Table 2) were selected as the nearest preceding non-overlapping time frame beginning on the same weekday as the ASDE.

**Figure 3:**
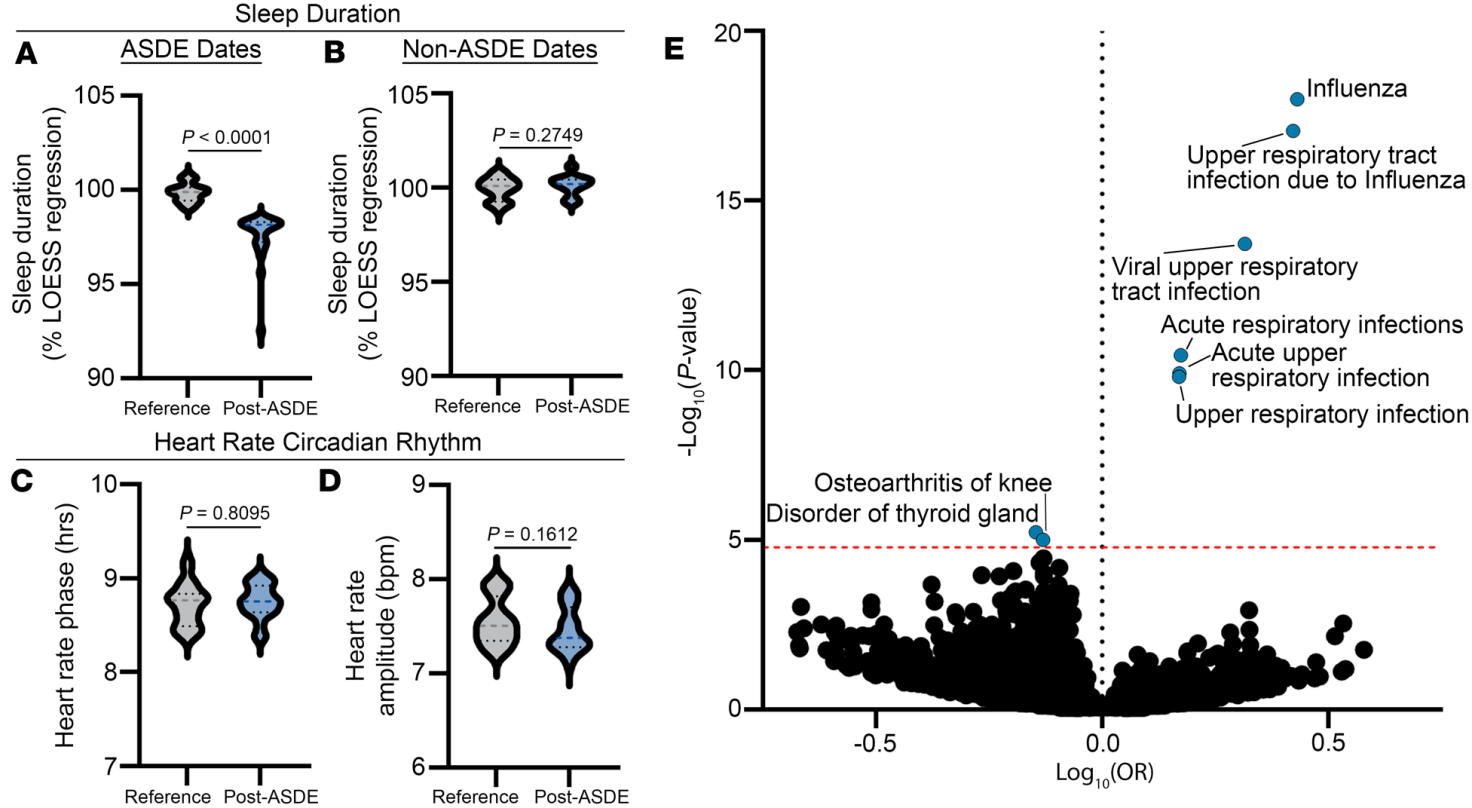
Influenza and acute respiratory infections are increased following ASDEs. (**A** and **B**) Sleep duration on the ASDE dates (**A**) and non-ASDE dates (**B**) of the post-ASDE periods and their corresponding dates in the reference periods as a percentage of the LOESS regression. (**C** and **D**) Phase (**C**) and amplitude (**D**) of circadian heart rate rhythms in reference and post-ASDE periods. Dashed line on violin plots is median; dotted lines are interquartile range. (**E**) Phenome-wide frequency of new diagnoses was compared between post-ASDE periods and reference periods by McNemar’s test in the AoU population (*n* = 287,012). Red dashed line represents Bonferroni-adjusted *P* value threshold. Diagnoses with significant (adjusted *P* value < 0.05) differences in odds ratio (OR) are annotated. Between-period sleep durations and heart rate amplitudes were compared by Mann-Whitney test. Heart rate phase was compared by non-parametric Wheeler-Watson test for circular data. Unadjusted (**A**–**D**) or adjusted (**E**) *P* < 0.05 was considered statistically significant.

We confirmed that the average sleep duration as a percentage of the LOESS-predicted sleep duration was significantly lower on the ASDE dates of the post-ASDE periods as compared with the corresponding dates of the reference periods (Figure 3A). Meanwhile, sleep duration was no different on the non-ASDE dates of the post-ASDE versus reference periods (Figure 3B), consistent with acute sleep deprivation. Because sleep deprivation can lead to circadian desynchronization (22, 23), we also examined the phase and amplitude of heart rate rhythms as an indicator of circadian timing. By this method, we did not detect a shift in circadian timing in the post-ASDE versus reference periods (Figure 3, C and D), highlighting sleep duration as a crucial difference between the periods.

Having defined our time periods for comparison, we next conducted a phenome-wide analysis comparing incident diagnoses, regardless of health care setting, occurring in the pooled post-ASDE periods versus reference periods by McNemar’s test. In this manner, we identified a significantly increased risk of influenza (1,052 vs. 683 incident cases, odds ratio [OR] = 1.54 [1.40, 1.70], *P* = 1.00 × 10^–18^) and other related respiratory conditions in the pooled post-ASDE period (Figure 3E and Supplemental Table 3). Based on this finding, we directed our attention to the association of ASDEs and influenza risk.

Because the duration of the ASDE effect is unknown, we only compared post-ASDE periods to preceding (as opposed to trailing) reference periods. However, to account for the strong seasonality of influenza incidence (24), we leveraged the fact that national political events tend to occur in 4-year cycles. In this regard, we compared the incident influenza diagnoses in the post-ASDE periods of national political events (Table 2) with their incidence in a reference period exactly 1 year later (Supplemental Table 4). Consistent with our hypothesis that ASDEs were associated with increased influenza risk, we observed an increased incidence of influenza-associated health care visits in the political post-ASDE periods as compared with the following year (166 vs. 111 incident cases, OR = 1.50 [1.17, 1.92], *P* = 0.0012). Additionally, it is possible that confounding factors such as travel, stress, and anxiety associated with national events and holidays drive the observed signal. However, the post-Thanksgiving holiday period, which was not an ASDE but may have similar characteristics to other major US holidays (Supplemental Table 4), did not have a significant association with incident influenza health care visits compared with the preceding reference period (123 vs. 106 incident cases, OR = 1.16 [0.89, 1.52], *P* = 0.2883). Finally, because the demographic composition of the Fitbit-wearing population differs in key respects from the overall population (18), we constructed a balanced cohort from the EHR population, which was matched to the Fitbit population along age, race, sex, time zone, and educational attainment (Supplemental Table 5). In this balanced cohort (*n* = 72,769 participants), we continued to observe an increased risk of incident influenza in the post-ASDE period (295 vs. 204 incident cases, OR = 1.45 [1.21, 1.74], *P* = 5.60 × 10^–5^). Taken together, these data suggest an association between ASDEs and influenza risk.

### Identification of short sleep genotypes.

**Figure 4:**
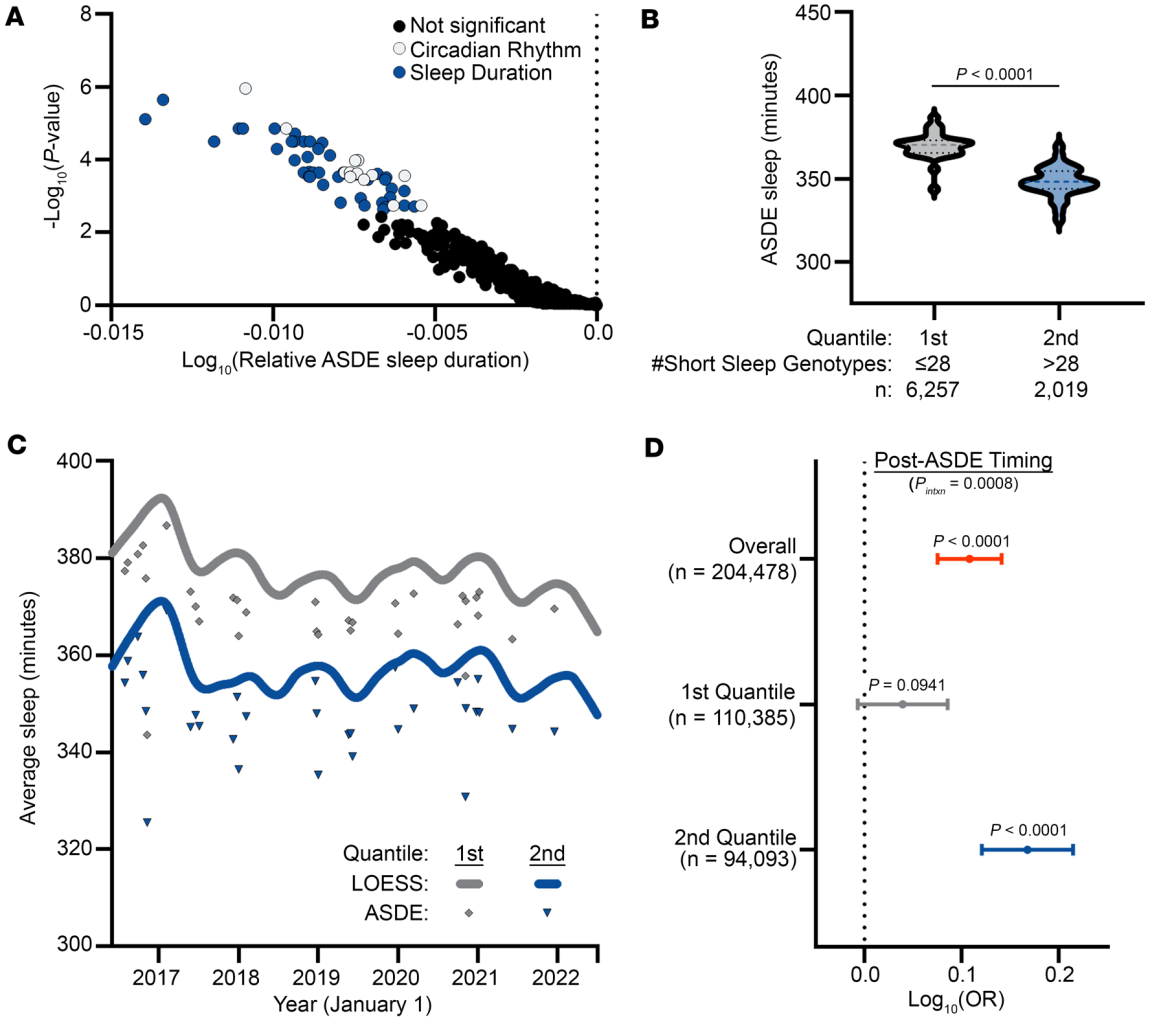
Circadian and sleep genotypes are associated with sleep duration and post-ASDE influenza risk. (**A**) Volcano plot of SNP allele combinations associated with shorter sleep duration on ASDEs. SNPs associated with significantly shorter sleep duration than the AoU population on ASDEs after correction for false discovery rate are highlighted. (**B** and **C**) Genotyped AoU participants were grouped into quantiles based on their number of short sleep–associated SNP allele combinations. Sleep duration in the genotyped AoU Fitbit population was decreased by short sleep genotype quantiles on ASDEs (**B**) and overall (**C**). (**D**) Forest plot of log_10_(odds ratio) of influenza diagnosis as a function of post-ASDE timing overall and grouped by short sleep genotype quantile with adjustment for age at ASDE, sex chromosome ploidy, time-weighted average weekly positive influenza tests, and genomic ancestry in a generalized estimating equation model. Comparisons in ASDE sleep duration between SNP genotypes as well as between ASDE sleep duration quantiles were performed using Mann-Whitney test. Dashed line on violin plot shows median; dotted lines show interquartile range. *P* < 0.05 was considered statistically significant. *P_intxn_*, interaction *P* value (short sleep genotype quantile × post-ASDE timing).

Given the apparent link between ASDEs and influenza risk, we hypothesized that individuals who are genetically predisposed to less sleep would be at greater risk of an influenza-associated health care visit after an ASDE. Misalignment of the circadian rhythm and the sleep homeostat, both of which are subject to genetic influence (25), contribute to human disease manifestation across a broad range of organ systems. Therefore, we hypothesized that genetic perturbations to the sleep homeostat modify the risk of post-ASDE incident disease. We first obtained lists of single-nucleotide polymorphisms (SNPs) associated with either circadian rhythm (Gene Ontology [GO]: 0007623) or sleep duration (Experimental Factor Ontology [EFO]: 0005271) from the publicly available genome-wide association study (GWAS) catalog (26). Using the genotyped segment of the AoU Fitbit cohort (*n* = 8,276), we identified common variants (minor allele frequency > 5% [ref. 27]; Supplemental Table 6) for further study. For every homozygous common variant genotype present in at least 20 AoU Fitbit participants, we quantified the average sleep duration on the 32 ASDEs as compared with the entire AoU Fitbit population. Through this approach, we identified 56 SNP genotypes associated with less ASDE sleep than the AoU population mean (Figure 4A and Supplemental Table 7), which we name “short sleep genotypes.” Participants were then binned into 2 quantiles according to their having at most (first quantile, *n* = 127,652) or greater than (second quantile, *n* = 115,828) the median number, 28, of short sleep genotypes. Heterozygous genotypes containing a significant allele were counted as 0.5 short sleep genotypes. We observed a significant decrease in ASDE sleep duration between short sleep genotype quantiles (Figure 4B); notably, bin proportions differed between the overall population and the Fitbit population because the median of the overall population was used in binning. Interestingly, there was also a shift in baseline sleep duration between genotype quantiles (Figure 4C), indicating that ASDE-associated sleep deficits take place in addition to genetically predisposed sleep durations.

### Synergistic interaction of ASDEs and sleep genotypes with incident influenza diagnoses.

Based on our findings of the dual influence of ASDEs and sleep genotypes on sleep duration, we hypothesized that ASDEs and sleep genotypes would interact to contribute to post-ASDE incident influenza. In the subset of the genotype AoU population with EHR data (*n* = 204,478), we used generalized estimating equations to calculate the OR of having a health care visit for influenza as a function of relative ASDE timing (before or after the ASDE), sleep genotype quantile, sex chromosome ploidy, genomic ancestry, and age on the date of the ASDE. To account for influenza seasonality, we also adjusted for the average weekly positive influenza tests, weighted by period length, based on public data from the US Centers for Disease Control and Prevention (28) (Supplemental Table 2). In our model, incident influenza-associated health care visits were significantly increased after ASDEs (OR = 1.28 [1.19, 1.39], *P* = 1.30 × 10^–10^) in the overall population (Figure 4D and Supplemental Table 8). In agreement with our hypothesis, the association of ASDEs with influenza incidence was heightened in individuals with a greater number of short sleep genotypes (OR = 1.47 [1.32, 1.64] vs. 1.10 [0.98, 1.22], *P* value of genotype-ASDE interaction = 0.0008; Supplemental Tables 9–11). Together, these findings suggest that population-level acute sleep deprivation and genetic predisposition jointly influence the risk of influenza incidence.

## Discussion

Here, we identified the spectrum of major national events associated with acute sleep deprivation in a heterogeneous sample of the American population. We demonstrated that these events, which we term ASDEs, are associated with increased incidence of influenza. We found that SNPs linked to circadian rhythm and sleep duration carry a cumulative influence on sleep duration and interact with ASDEs to jointly increase incident influenza. Taken together, this study offers needed insight into both predisposition to and health consequences of acute sleep deprivation across the American public. From a public health perspective, these findings may aid in predicting future events when sleep duration is jeopardized and when the American population is vulnerable to detrimental health outcomes.

Influenza is a recurring public health concern with between 9 million and 40 million cases occurring each year in the United States alone (29). Based on our findings, political events, holidays, and major sporting competitions are likely to be ASDEs where an increased risk of influenza could now be predicted. Our findings should strengthen the vigilance across health care systems for increased influenza cases in these periods, and mitigation plans could include increased flu vaccination campaigns. Importantly, sleep deprivation has been associated with diminished antiviral immunity following influenza vaccination (8, 30), suggesting a benefit of improved sleep hygiene proximal to vaccination as well. These efforts may be particularly beneficial for females, who, consistent with prior studies (31), were at increased influenza risk across all time periods in our model (Supplemental Table 11). If future studies confirm the association of genotype with sleep duration and influenza, increased genotyping of the American public could be useful to identify those at greatest risk on future ASDEs. We do not know whether similar results would be seen in other geographic locations and countries with potentially distinct ASDEs and sociopolitical interests.

Chronic sleep deprivation has been previously associated with the risk of influenza and viral upper respiratory infections (32–36). However, this study is the first to our knowledge to identify an association across a large and heterogeneous population between acute sleep deprivation and influenza risk. We found a robust difference in ASDE sleep duration based on an individual’s quantity of sleep and circadian-associated allelic variants, which builds upon a prior observation of the additive influence of sleep duration genotypes in a cohort with 7 days of actigraphy data (37). Our observation that genotypes associated with less habitual sleep duration are also prone to less ASDE sleep duration suggests that the relationship between chronic sleep deprivation and influenza risk may be explained by the effects of acute-on-chronic sleep duration rather than habitual sleep duration alone. In fact, our model of ASDE and genotype interaction suggests that sleep genotypes themselves are not significantly associated with influenza risk; rather, our model implies that individuals with short sleep genotypes are more vulnerable to the effects of ASDEs on influenza risk (Supplemental Table 11).

Here, we focused on acute health events in the immediate aftermath of a single night of sleep deprivation. We only examined first-time diagnoses occurring in a 10-day window following ASDEs, a duration that was chosen to allow for the selection of preceding reference periods during the study period and limit overlapping post-ASDE periods. Influenza has a reported incubation time of 1 to 4 days (38), and prior meta-analyses have suggested that 75% of hospitalized influenza patients present within 5 days of symptom onset (39), suggesting the appropriateness of this window for influenza. However, it is possible that ASDEs also pose long-term risks that could, for example, contribute to the proposed effects of major political events on mental health (40). Longitudinal analyses of the AoU program will be crucial to defining these types of outcomes. Prior studies have also reported an association between major sporting and political events (41–43) and acute cardiovascular disease. While our unbiased phenomic analysis of the comparatively small AoU cohort did not identify a link to cardiovascular disease, the presence of similar types of events among ASDEs raises the question of whether acute sleep deprivation may be contributing to cardiovascular disease as well.

We acknowledge limitations to this work. This study is subject to selection bias of the AoU program, and ASDEs identified in AoU’s Fitbit subset may not reflect those of the larger AoU population, thereby limiting generalizability. In addition, while Fitbit-based sleep estimates enable population-level analyses of sleep and health (17, 18), they are subject to limitations and are not a substitute for gold standard polysomnography or medical-grade actigraphy (44). Although our study demonstrates a connection between ASDEs and influenza, this finding remains associative. There was substantial overlap between the study period and the COVID-19 pandemic, which may limit the generalizability of our findings to inform future policy (45). Our study is limited to the analysis of incident influenza-associated health care visits, which may reflect increased infection rate, greater symptom burden, or greater likelihood to seek medical attention following an ASDE. The ASDE-influenza relationship is further confounded by the fact that stress, substance use, or other unmeasured confounders may be increased around ASDEs (46, 47). Given that ASDEs coincide with major national events, it is possible that large social gatherings rather than sleep deprivation are driving the observed increases in influenza. Thanksgiving, by comparison, is associated with large gatherings in close seasonal proximity to elections but was not an ASDE; the absence of a similar increase in influenza after Thanksgiving, therefore, suggests against a major confounding contribution from large gatherings. Because our study was limited to weekdays, some potential ASDEs, including daylight savings time, were not examined. Our incorporation of sleep genotypes strongly suggests a direct link between sleep deprivation and influenza risk, although determination of causality requires further proof. Given that sleep duration constitutes only one part of sleep health (48), future studies should also incorporate metrics of sleep architecture beyond sleep duration.

In conclusion, this study defines the population-level landscape of ASDEs in the United States, identifies an acute association between ASDEs and influenza risk, and incorporates sleep genomics to suggest a direct link between acute sleep deprivation and influenza. We propose that these findings should guide health system preparations and public health activities around major national events.

## Methods

### Sex as a biological variable.

This study included all sexes. Sex was considered as a biological variable and adjusted for in regression models.

### All of Us.

This study used data from the AoU Research Program’s Controlled Tier Dataset 7. The study period encompassed June 1, 2016, through July 1, 2022. No participants were excluded from the study.

### ASDE determination.

Daily weekday minutes slept were averaged for AoU participants with available sleep data. Weekend nights, which are associated with longer sleep duration than weekday nights, were excluded from the analysis (49). Locally estimated scatterplot smoothing (LOESS) regression of minutes slept, as well as confidence intervals, was generated using the Python package tsmoothie v1.0.5 (https://github.com/cerlymarco/tsmoothie). with a smoothing fraction of 0.1 and one iteration. For 1-sided analysis of sleep deprivation, dates with average minutes slept less than the 90% confidence interval of the LOESS regression were considered ASDEs.

### Demographic reporting.

Demographic categories were defined by the AoU research program and selected by the participants. For reporting purposes, sex categories (intersex) and race categories (Middle Eastern or North African, Native Hawaiian or Other Pacific Islander, and more than one population) with data from less than 20 participants on any ASDE were pooled with unknowns in accordance with AoU requirements.

### Television viewership.

Television viewership data were obtained from archived online reports of Nielsen cable and national broadcast ratings (20).

### Google Trends.

Google Trends is an online tool that records archival internet search volume (19). The top 3 Google Trends search queries in the United States were annotated for the day prior to each ASDE.

### Time zones.

Five-digit US ZIP code coordinates were obtained from the US Census Bureau ZIP Code Tabulation Areas Gazetteer File (50) of 2020 Census tabulation blocks. In order to identify coordinates for the 3-digit ZIP codes supplied by the AoU Controlled Tier Dataset, the constituent 5-digit ZIP code coordinates (latitude and longitude) were averaged. Time zones of the resultant coordinates were obtained from the Python implementation of Google Maps (googlemaps v4.10.0).

### State political partisanship.

The Cook Partisan Voting Index (Cook PVI) measures a state’s lean toward the Republican (R) or Democratic (D) Party in US presidential elections (e.g., R+1 indicates a 1-percentage-point Republican lean). The 2022 Cook PVI was obtained from publicly available data (51). For the generation of quartiles, leans were expressed as negative or positive numbers depending on party.

### Time-weighted average positive influenza tests.

National weekly positive influenza tests (influenza A and influenza B) from clinical laboratories were obtained from the US Centers for Disease Control and Prevention’s FluView tool for the 2016–2017 through 2021–2022 seasons (28). Positive tests were averaged over all seasons by week of the year. The average positive tests corresponding to a time period were determined by averaging the weekly positive tests for the week of the year associated with each day of the period and multiplying by the number of weeks in the period.

### Post-ASDE and reference period selection.

Post-ASDE periods were defined as the 10 days beginning on the ASDE. If a post-ASDE period overlapped with a subsequent post-ASDE period, they were pooled into a combined period beginning on the earliest of the overlapping ASDEs and ending on the tenth day of the latest ASDE period. Reference periods were chosen for each pooled post-ASDE period as the latest period of the same duration beginning on the same weekday as its corresponding post-ASDE period that did not overlap with a post-ASDE period. For the separate comparison of political post-ASDEs, reference periods were selected as the period of the same duration beginning on the same weekday as its corresponding post-ASDE period that was closest to 365 days after the corresponding ASDE.

### AoU diagnoses.

Data were organized and annotated using the Observational Medical Outcomes Partnership common data model, which classifies health conditions according to a hierarchical tree (52). Conditions occurring within post-ASDE or reference periods were considered. A condition was considered to have occurred if either the condition or one of its first-degree descendants occurred in a period. For the phenome-wide analysis, conditions that had been previously associated with the participant before the period start date were excluded so as only to examine new (incident) diagnoses. In order to focus our search on clinical disorders rather than symptoms, we limited our analysis to conditions with an ancestor of (a) “mental disorder” (ID 432586) or (b) “disorder of body system” (ID 4180628) and either “disorder of nervous system” (ID 376337), “disorder of endocrine system” (ID 31821), “disorder of digestive system” (ID 4201745), “disorder of cardiovascular system” (ID 134057), “disorder of musculoskeletal system” (ID 4244662), “disorder of the genitourinary system” (ID 4171379), “disorder of respiratory system” (ID 320136), “disorder of auditory system” (ID 4176644), “disorder of lymphatic system” (ID 440363), “red blood cell disorder” (ID 432739), “hereditary disorder by system” (ID 4180158), or “visual system disorder” (ID 4134440). For analyses of influenza specifically, including analysis of political events, Thanksgiving, and generalized estimating equations, we allowed for the possibility that an individual may contract influenza in multiple years; therefore, the diagnosis of influenza was considered to have occurred in a given period if it was diagnosed in that period and had not been diagnosed in the preceding 180-day period.

### Matched cohort construction.

Participants with EHR data (*n* = 287,012) were matched 5:1 with Fitbit (Google) participants (*n* = 14,681) according to age (binned to 5-year intervals), race (White, Black or African American, Asian, or other/unknown), sex at birth, college education, and time zone using the Python implementation of Dynamic Almost Matching Exactly (DAME) in dame-flame (v0.81). As a result, 14,525 Fitbit participants (98.94%) were fully matched, 60 participants (0.41%) were partially matched, and 96 participants (0.65%) were unmatched, with a final matched cohort size of *n* = 72,769 participants.

### Sleep deprivation comparison.

Daily minutes slept were smoothed using LOESS regression with a smoothing fraction of 0.1 and one iteration for each subgroup (e.g., male and female, political lean quartile). Sleep deficit was calculated as the difference between the subgroup’s mean sleep duration and its LOESS fit for each ASDE. Comparisons between subgroups were performed by Mann-Whitney test (2 subgroups) or Kruskal-Wallis test with Dunn’s multiple-comparison test (3 or more subgroups) using GraphPad Prism.

### Single-nucleotide polymorphisms.

SNPs associated with circadian rhythm (*N* = 133 total, *n* = 126 in AoU genomics records) and sleep duration (*N* = 960 total, *n* = 895 in AoU genomics records) were obtained from the GWAS catalog (26) using the trait identifiers GO_0007623 and EFO_0005271, respectively. Genotypes, sex ploidy, and genomic ancestry were obtained from the AoU workbench (53). Minor allele frequencies (MAFs) were calculated for each SNP allele based on their frequencies in the AoU genomics population (*n* = 243,480). Alleles with MAF greater than 5% and with homozygosity in at least 20 participants in the AoU genotyped Fitbit population (*n* = 1,323 alleles) were included.

### Heart rate phase.

Average heart rate was aggregated over 5-minute intervals for all participants with available data. Average 5-minute heart rate during the reference of post-ASDE period was fit to a sine curve of the form where *H* is heart rate, *A* is amplitude, *t* is time in minutes from the beginning of the respective reference or post-ASDE period, *φ* is heart rate phase, and *b* is baseline shift. Heart rate phase and amplitude were averaged for all individuals with heart rate data available on more than 90% of time points in each (post-ASDE or reference) period. Statistical comparisons of circular mean heart rate phase during reference or post-ASDE periods were made using the Wheeler-Watson test in the Python module pycircstat2 v0.1.12. Comparisons of heart rate amplitude were made by Mann-Whitney test.

### Phenome-wide odds ratio analysis.

Post-ASDE and reference periods were pooled to generate a single combined post-ASDE or reference period such that overlapping time frames were not double-counted. Diagnoses corresponding to fewer than 20 participants in either the combined (corresponding to all ASDEs) reference periods or ASDE periods were excluded per AoU data use policy. Comparisons of condition instances between time periods were conducted using the mcnemar implementation of McNemar’s test in the SciPy package (v1.11.2) (54). OR was estimated as the number of post-ASDE instances divided by the number of reference instances. The SciPy binomial test binom_test was used to calculate 95% confidence intervals. Bonferroni-adjusted *P* values less than 0.05 were considered statistically significant for the phenome-wide analysis.

### SNP sleep duration comparison.

Average ASDE sleep duration was compared with that of the AoU population using the Mann-Whitney test (mannwhitneyu function of the Python package SciPy). The SciPy bootstrap function with default parameters was used to calculate 95% confidence intervals. Multiple test correction was performed by the Benjamini-Hochberg procedure (SciPy method false_discovery_control) with a false discovery control level of α = 0.05. Homozygous SNP genotypes with a sleep ratio (vs. AoU population) less than 1.0 and adjusted *P* value less than 0.05 were considered short sleep genotypes. Genotyped AoU participants were assigned to quantiles based on their number of short sleep genotypes using the pandas qcut function in Python with 2 groups centered on the population median (*n* = 243,480). Heterozygous genotypes containing a significant allele were counted as 0.5 short sleep genotypes. ASDE sleep duration comparison between quantiles was performed by Mann-Whitney test in GraphPad Prism.

### Generalized estimating equations.

Generalized estimating equations of the binomial family distribution with logit link function, exchangeable dependence structure, and robust standard errors were used to calculate relationships between hospitalizations, time period (pre- or post-ASDE), short sleep genotype quantile, sex chromosome ploidy, genomic ancestry, average positive influenza tests, and age at ASDE. Covariates were selected based on prior knowledge (31, 55, 56). Generalized estimating equation analysis was performed using Statsmodels v0.14.2 (57).

### Statistics.

Summary statistics were plotted in Prism v10.2.0 for Windows (GraphPad Software). Statistical tests were performed in Python v3.10.12 or GraphPad Prism as specified. A *P* value of less than 0.05 was considered statistically significant unless otherwise noted.

### Study approval.

All participants provided written informed consent to the AoU Research Program. In accordance with the AoU Research Program, this study was exempt from human subject approval, and only deidentified data were analyzed.

### Data availability.

AoU data are available to authorized users of the AoU Research Program’s Controlled Tier Dataset 7 on the Researcher Workbench at https://www.researchallofus.org/ Values for all data points in graphs are reported in the Supporting Data Values file.

## Author contributions

NJK, RC, WEK, and SYC conceived the project and wrote the manuscript. NJK, WEK, NK, JW, PP, INC, HR, AS, and ANS analyzed the data and prepared the figures. SO, CAM, SMN, and SYC supervised the project. All authors reviewed, edited, and approved the manuscript. The order of the co–first authors’ names was assigned according to the academic contribution of each author.

## Funding support

This work is the result of NIH funding, in whole or in part, and is subject to the NIH Public Access Policy. Through acceptance of this federal funding, the NIH has been given a right to make the work publicly available in PubMed Central.

- WoodNext Foundation (to SYC and CAM).
- NIH grants T32HL129964 (to NJK and RC), K08ES037420 (to NJK), R01HL124021 (to SYC), R01HL122596 (to SYC), and R01HL151228 (to SYC).
- American Heart Association grants 24SFRNCCN1276089 and 24SFRNPCN1280228 (to SYC).
- United Therapeutics Jenesis Innovative Research Awards (to NJK).
- Pulmonary Hypertension Association (to NJK).
- McKamish Family Foundation Cardiovascular Innovator Awards (to NJK and RC).

## Supplementary Material

Supplemental data

ICMJE disclosure forms

Supplemental tables 1-11

Supporting data values

## Figures and Tables

**Table 1:**
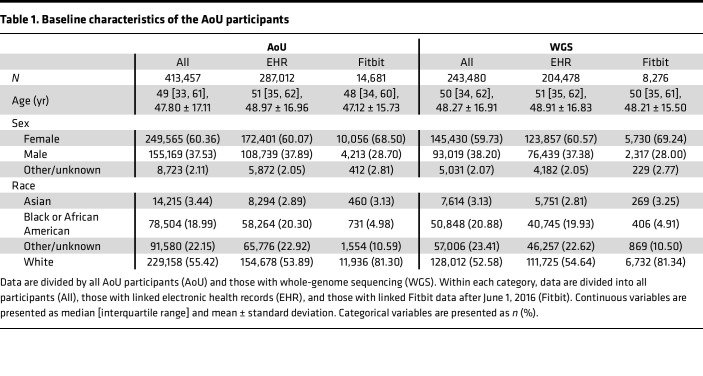
Baseline characteristics of the AoU participants

**Table 2:**
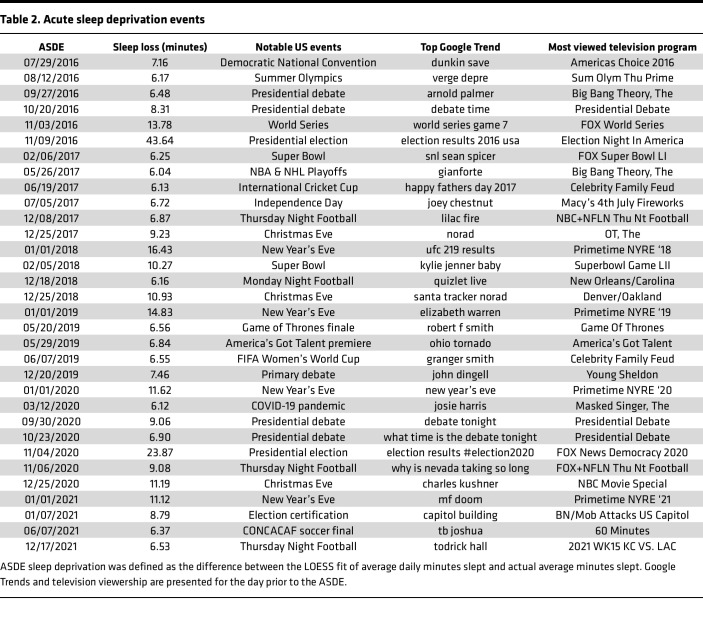
Acute sleep deprivation events

## Version 1 12/23/2025

In-Press Preview

## Version 2 02/09/2026

Electronic publication
