# Supplementary material for: Health outcomes after national acute sleep deprivation events among the American public
Source: JCI Insight. 2025 Dec 23;11(3):e195344. doi: 10.1172/jci.insight.195344 (PMC12892888; doi:10.1172/jci.insight.195344)

Health outcomes after national acute sleep deprivation events among the American public

Neil J. Kelly[1,2,7,*,†], Rahul Chaudhary[1,2,7,*], Wadih El Khoury[1,2,*], Nishita Kalepalli[1,2], Jesse Wang [1], Priya Patel[1,8], Irene N. Chan[1], Haris Rahman[1], Aisha Saiyed[9], Anisha N. Shah[1,2], Colleen A. McClung[4], Satoshi Okawa[1,2,5,6], Seyed Mehdi Nouraie[1,3], Stephen Y. Chan[1,2,†]

[1]Center for Pulmonary Vascular Biology and Medicine, Pittsburgh Heart, Lung, and Blood Vascular Medicine Institute, [2]Heart and Vascular Institute, [3]Division of Pulmonary, Allergy, and Critical Care Medicine, Department of Medicine, [4]Translational Neuroscience, Department of Psychiatry, [5]Department of Computational and Systems Biology, [6]McGowan Institute for Regenerative Medicine, University of Pittsburgh School of Medicine and University of Pittsburgh Medical Center, Pittsburgh, PA, USA. [7]Pittsburgh VA Medical Center, Pittsburgh, PA, USA. [8]Wellesley College, Wellesley, MA, USA. [9]University of California Davis, Davis, CA, USA. *Drs. N. Kelly, R. Chaudhary, and W. Khoury contributed equally to this manuscript.

[†]Correspondence:
Neil J. Kelly, MD, PhD
University of Pittsburgh School of Medicine
1704 Biomedical Science Tower, 200 Lothrop Street, Pittsburgh, PA 15261
Phone: (412) 383-6990, Email: njk88@pitt.edu

Stephen Y. Chan, MD, PhD (**contact**)
Pittsburgh Heart, Lung, and Blood Vascular Medicine Institute
University of Pittsburgh School of Medicine and UPMC
E1240 Biomedical Science Tower, 200 Lothrop Street, Pittsburgh, PA 15261
Phone: (412) 383-6990, Email: chansy@pitt.edu

List of Supplemental Tables. Tables are provided in a separate Excel file.

Supplemental Table 1. Acute sleep deprivation events with top 3 Google Trends and television broadcasts of the prior day. P2+: People ages 2 and up from Nielsen ratings.

Supplemental Table 2. ASDE and reference dates populated with average weekly positive flu tests weighted for block length.

Supplemental Table 3. Phenome-wide analysis of new diagnoses in the 10-day period following the pooled ASDEs. P-value was calculated from McNemar's test. P-value < 1.65×10^{-5} (0.05/3,036) was considered statistically significant.

Supplemental Table 4. Event dates and reference dates for political ASDEs and Thanksgiving.

Supplemental Table 5. Participant characteristics in the Fitbit, Matched EHR, and Total EHR cohorts. Participants from the Total EHR cohort were matched up to 5 to 1 with Fitbit participants along age, sex, race, time zone, and education to select the Matched EHR cohort. Data are mean ± standard deviation or n (%).

Supplemental Table 6. Sleep and circadian common variants.

Supplemental Table 7. ASDE sleep deprivation genotypes. Sleep ratio is average ASDE minutes slept in genotype versus All of Us population. P-values were calculated by Mann Whitney test and adjusted for false discovery rate by Benjamini-Hochberg method. Adjusted P-value < 0.05 was considered statistically significant.

Supplemental Table 8. Odds ratios and confidence intervals of influenza healthcare visit as a function of post-ASDE time period adjusted for time-weighted average weekly positive influenza tests, age, sex ploidy, and genomic ancestry prediction in a zero-intercept generalized estimating equation model (n = 204,478).

Supplemental Table 9. Odds ratios and confidence intervals of influenza healthcare visit in short sleep genotype quantile 1 (n = 110,385) as a function of post-ASDE time period adjusted for time-weighted average weekly positive influenza tests, age, sex ploidy, and genomic ancestry prediction in a zero-intercept generalized estimating equation model.

Supplemental Table 10. Odds ratios and confidence intervals of influenza healthcare visit in short sleep genotype quantile 2 (n = 94,093) as a function of post-ASDE time period adjusted for time-weighted average weekly positive influenza tests, age, sex ploidy, and genomic ancestry prediction in a zero-intercept generalized estimating equation model.

Supplemental Table 11. Odds ratios and confidence intervals of influenza diagnosis as a function of short sleep genotype quantile*post-ASDE time period adjusted for time-weighted average weekly positive influenza tests, age, sex ploidy, and genomic ancestry prediction in a zero-intercept generalized estimating equation model (n = 204,478).

Supplemental Figure Legends:

Supplemental Figure 1. *Population sleep duration during post-ASDE periods.* Mean sleep duration was plotted as a percentage of LOESS regression-predicted sleep duration for weeknights of each post-ASDE period (**A-W**). Axes are labeled with the first and last date of each post-ASDE period with ticks at 1-day intervals. Error bands show plus or minus 1 standard deviation. ASDE dates are highlighted in red. Dark blue gaps denote weekends.

Supplemental Figure 1.

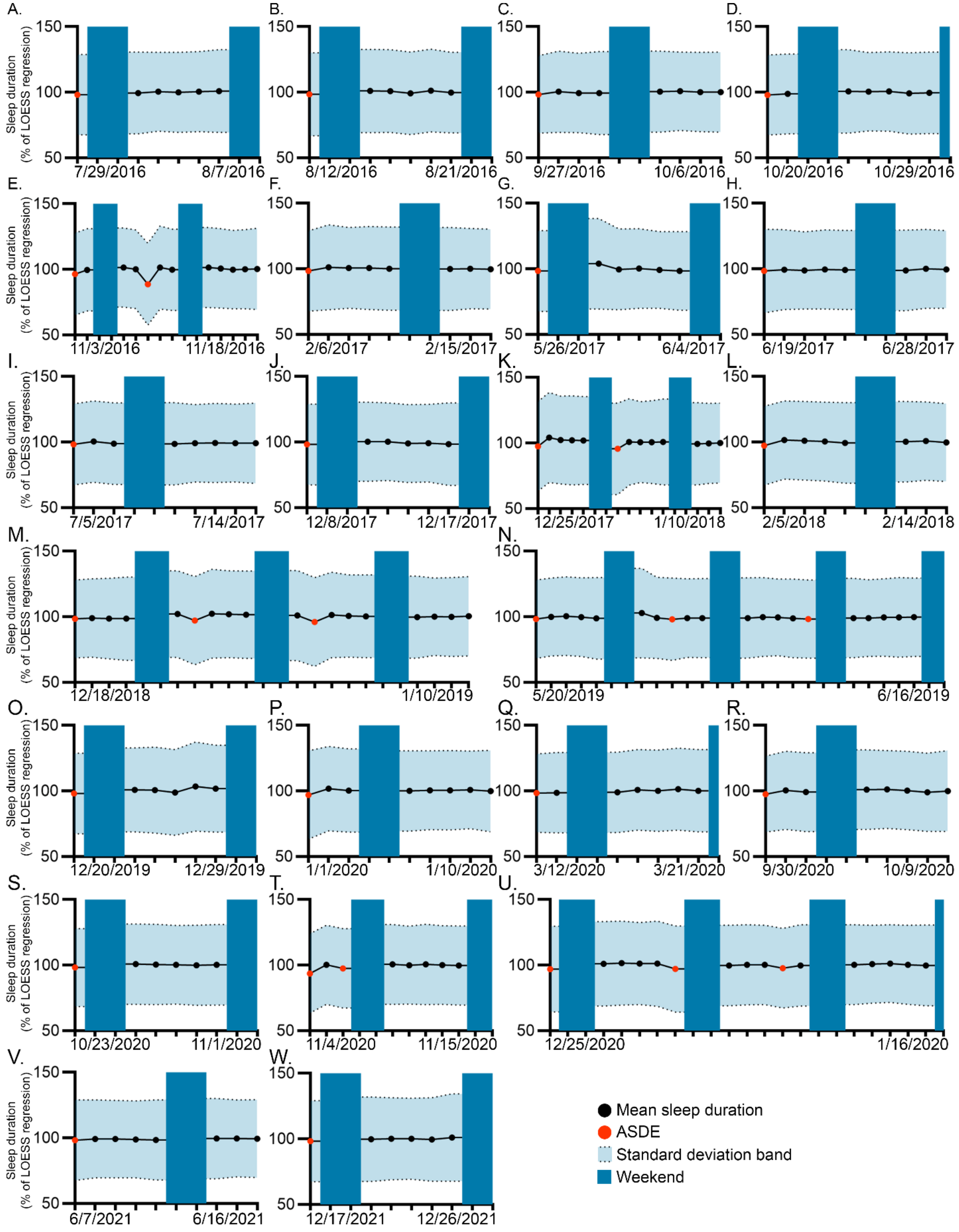

Supplement: Supplemental data [file jciinsight-11-195344-s125.pdf]
